# Comparative Assessment of Cephalometric and Tympanometric Readings in Down Syndrome

**DOI:** 10.7759/cureus.3301

**Published:** 2018-09-13

**Authors:** Sunali Khanna, Prita A Dhaimade, Rangasayee Raghunathrao

**Affiliations:** 1 Municipal Corporation of Greater Mumbai, Nair Hospital Dental College, Mumbai, IND; 2 Oral and Maxillofacial Surgery, Cooper Hospital, Mumbai, IND; 3 Hearing and Speech Sciences, Dr. S. R. Chandrasekhar Institute of Speech and Hearing, Bengaluru, IND

**Keywords:** down syndrome, cephalometry

## Abstract

Aim

The purpose of this study was to conduct a comparative assessment of the various cephalometric and auditory parameters between patients with Down syndrome (DS) and healthy controls.

Methods

The cephalometric and auditory parameters were divided among 50 participants into two equal sets, DS (*n *= 25) and controls (n=25), and assessed. While a standard cephalometric analysis was conducted to measure the hard tissue parameters, tympanometry was used to assess the audiological parameters.

Results

The values of the linear and angular cephalometric parameters of the DS group were found to be lower than the controls. All the controls recorded type A tympanogram while the DS group recorded type A, B, and C tympanograms. A significant relationship was observed in the cephalometric readings – eustachian tube (ET) length, posterior upper facial height (PUFH) length, sella (s)-basion (ba)-palatal length (PL), and s-ba-ET angles – among the subjects who presented with type B or C tympanogram in comparison to those with type A.

Conclusion

Tympanometry, a highly sensitive and relatively simple test to assess audiological parameters, has a significant relationship with a number of cephalometric indicators of growth and development. A deviation from the normal tympanometric readings can be used as an early indicator of the impending craniometric aberrations and handicap. This can be used as an effective tool for early intervention in cases of DS. Patients who have recorded abnormal tympanograms on multiple occasions over a period of six months can be subjected to a further cephalometric analysis.

## Introduction

Down syndrome (DS) is the most common genetic cause of moderate intellectual disability in children [1]. It was first described by John Langdon Down, a British physician, in 1866. The incidence of DS is seen to be as high as one in every 800-1200 births, irrespective of race and inheritance. Genetically, it can occur as a result of mosaicism, translocation, and, most commonly, trisomy 21 (95%). In trisomy 21, the zygote contains three copies of chromosome 21, causing every cell to have 47 chromosomes. One of the major risk factors for this non-disjunction to occur is increasing maternal age [1]. DS has been associated with a number of pathological abnormalities, primarily of the craniofacial region, including brachycephaly, reduced oral fissure, dental abnormalities, macroglossia, flat nasal bridge, etc., and several audiological deficiencies [2].

With the evolution of modern medicine, the average life expectancy of a child with DS has increased significantly [3]. With a better understanding of the pathophysiology of this condition, the focus has shifted from merely increasing life expectancy to providing a better quality of life to a child with DS [3]. A number of studies have explored the relationship between a child’s audiological abilities and normal craniofacial and intellectual development along with the importance of early intervention [4]. Language and speech development occurs in children primarily before the age of five years, and any impairment in this can lead to multiple, long-term developmental disabilities. The otolaryngologic and audiological impairments in DS are not uncommon and hence create a potential of additional disability [5]. Many anomalies of the pinna and middle ear have been noted in DS along with hypoplasia of the epitympanum and the round window, ossicular abnormalities and, most importantly, structural abnormalities of the eustachian tube (ET) [5]. The acute angle of entry into the nasopharynx, along with the diminished tube size classically seen in DS predispose the patient to recurrent infections of the middle ear, ultimately increasing the risk of chronic otitis media. The incidence of chronic otitis media was found to be significantly higher in DS (39% to 89%) than in the general population (2.5%) [6-7].

Cephalograms and cephalometric analyses have been used as the standard measures of craniofacial growth and development. Along with orthodontic and skeletal growth analysis, lateral cephalograms are often employed to calculate various cranial parameters, such as ET length, total cranial base (TCB), posterior upper facial height (PUFH), maxillary depth (MD), etc. The calculation/monitoring of the audiological parameters and the middle ear status can be conducted in a non-invasive way using tympanometry and pure tone audiometry [8].

Early identification and intervention coupled with a regular re-evaluation of the audiological parameters before the onset of chronic otitis media can help reduce the severity of disability in the risk groups [9]. A multi-disciplinary approach must be adopted, which involves a dentist, an otolaryngologist, a speech therapist, etc., along with the consulting pediatrician, to improve the social and cognitive function of children with DS, thus ensuring a bridging of the gap toward a normal life [7]. The aim of this study was to assess and explore the relationship between the cephalometric and audiological parameters of patients with DS and healthy controls.

## Materials and methods

A randomized convenience sampling technique was utilized for sample size determination. A total of 50 subjects were selected from the age group of seven to 20 years. Approval from the institutional ethical committee was obtained along with informed consent from all subjects and/or guardians prior to the commencement of the study.

The subjects were divided into two groups: DS (*n *= 25; determined using genotyping records) and healthy controls (*n *= 25).

Digital lateral cephalometry was carried out identically among all the subjects. They were exposed in an erect position with teeth occluded, lips in response, and head stabilized with a cephalostat. Radiation protection parameters were adhered to. Cephalometric tracing was carried out manually. Linear and angular measurements were recorded and tabulated.

**Figure 1:**
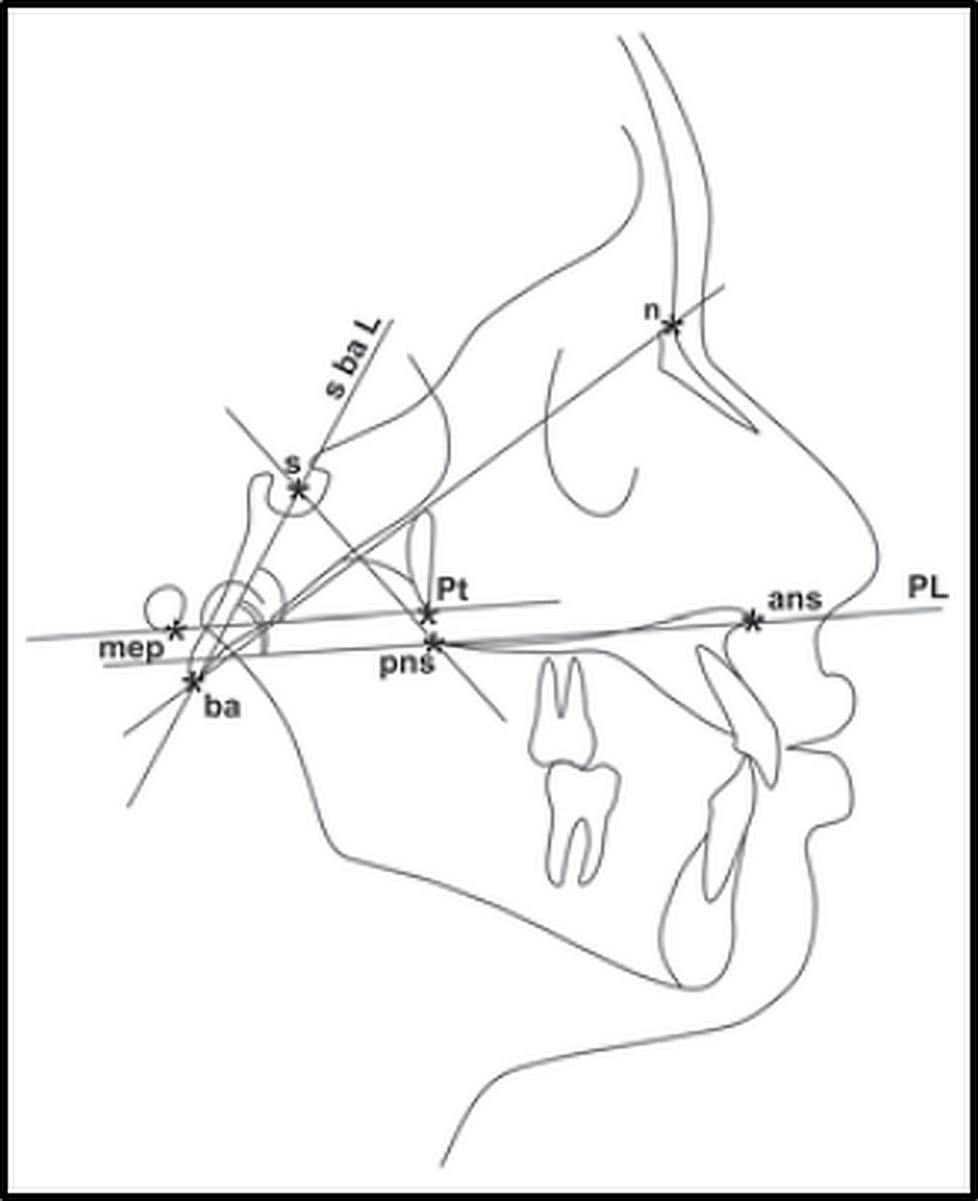
Cephalometric landmarks, linear and angular measurements s: sella, ba: basion, n: nasion, pns: posterior nasal spine, ans: anterior nasal spine, PL: palatal line

The craniofacial landmarks included were the following (Figure 1):

a. Linear measurements

i. ET length (mep-pt)

ii. TCB (nasion (n)-basion (ba))

iii. PUFH (sella (s)-posterior nasal spine (pns))

iv. MD ((anterior nasal spine (ans)-(pns))

b. Angular measurements

i. s-ba to palatal line (PL)

ii. s-ba to ET length

Tympanometry was the tool used to assess the audiological parameters among both groups. Further, both groups were subjected to ENT and audiological examinations were conducted by the Rehabilitation Council of India-certified audiologists. These tests were conducted in standard audiometric sound-treated rooms using standardized equipment calibrated as per the American National Standards Institute (ANSI): S3.6 -1996 specifications or the Bureau of Indian Standards (BIS) specifications IS 10565:1999 (R 2005) for diagnostic audiometers. The data were subjected to statistical analyses using Microsoft Office Excel (Microsoft Corporation, Redmond, WA, USA) and Statistical Package for Social Sciences (SPSS, IBM Corp., Armonk, NY, US) software. The confidence level chosen was 95%. The analysis of variance (ANOVA) test was used for a statistical analysis. 

## Results

**Figure 2:**
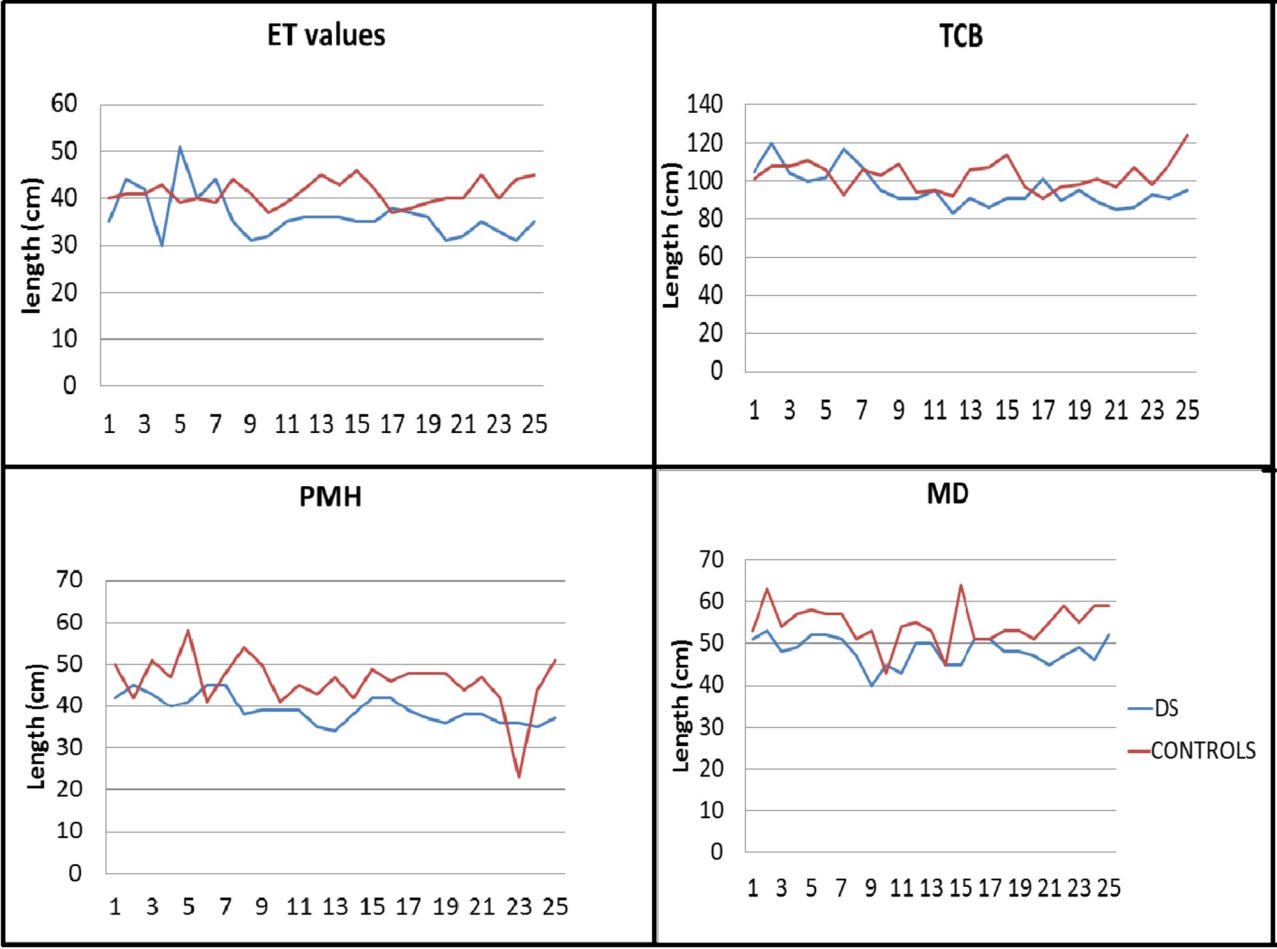
Comparison of linear cephalometric parameters among DS and controls ET: eustachian tube, TCB: total cranial base, PMH: posterior maxillary height, MD: maxillary depth, DS: Down syndrome

**Figure 3:**
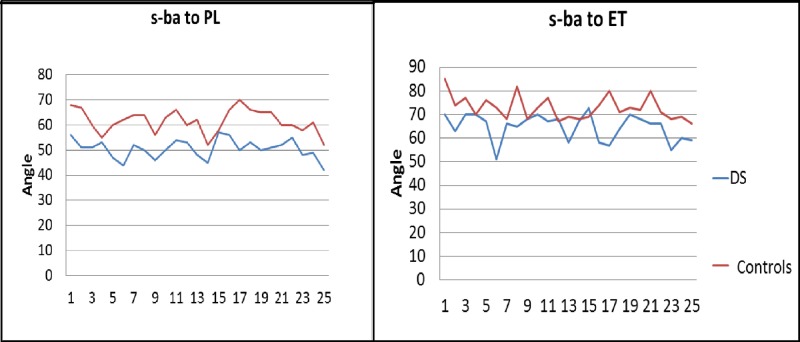
Comparison of angular cephalometric measurements among DS and controls s: sella, ba: basion, PL: palatal line, ET: eustachian tube, DS: Down syndrome

Standard cephalograms were recorded for both the controls and the DS group, followed by accurate cephalometric tracing and documentation of the readings. Figures 2-3 show a graphical representation of the linear and angular cephalometric readings of the controls and the DS group.

**Figure 4:**
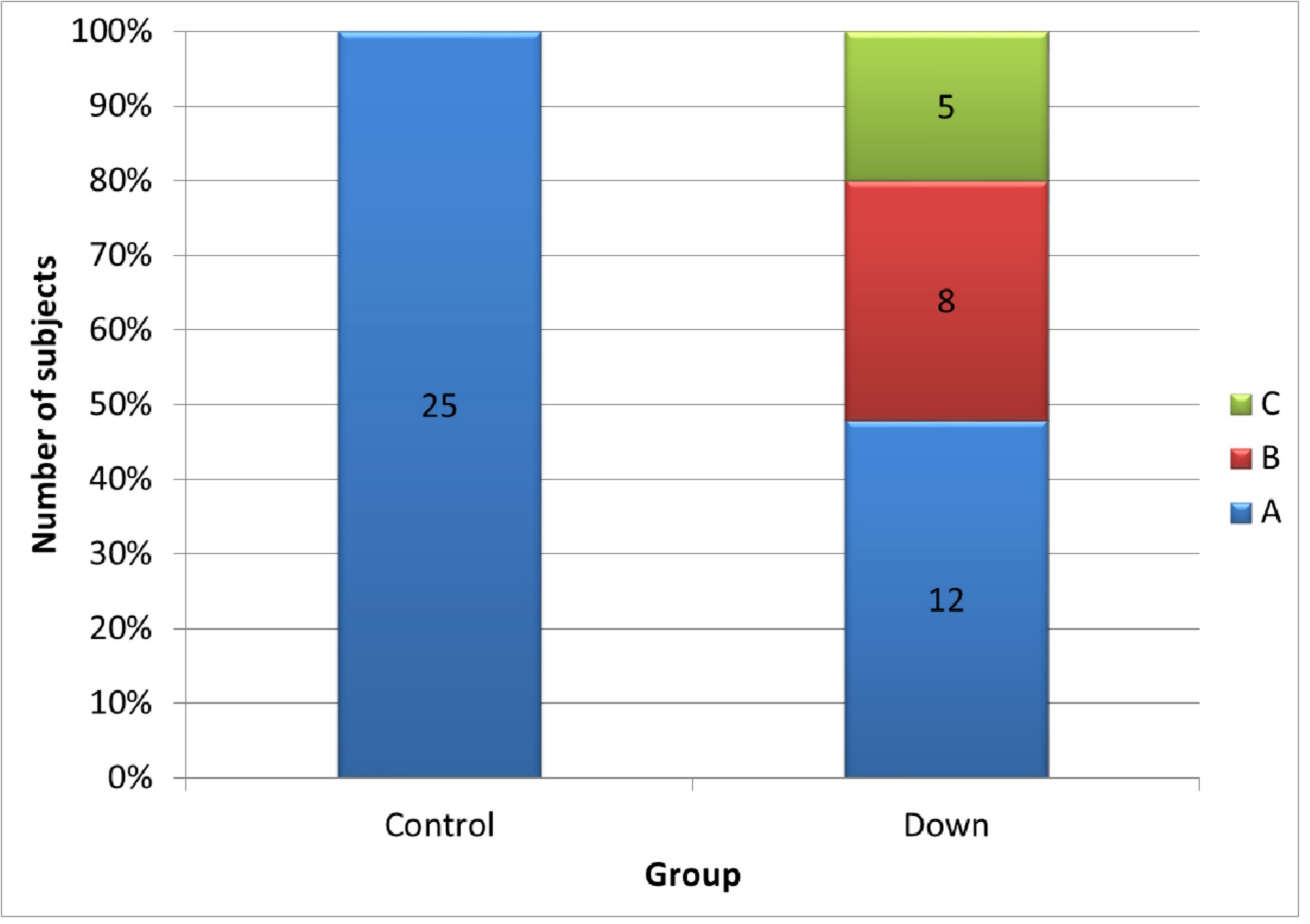
Tympanometric assessment of the DS group and the controls DS: Down syndrome

All controls presented with a 'type A' tympanogram while the DS group presented with 'type A, B, and C' tympanogram readings, as seen in Figure 4 and Table 1. When comparing A, B and C, the ‘*p*-value’ was found significant for two of the linear parameters, i.e., ET length and PUFH length, and both angular parameters, i.e., s-ba to PL and s-ba to ET length. Tables 2-5 represent statistical calculations for the same.

**Table 1:** Tympanometric assesment in DS and controls

Tympanogram	Control		Down		Total
	N	%	N	%	
A	25	100	12	48	37
B	0	0	8	32	8
C	0	0	5	20	5
Total	25	100	25	100	50

**Table 2:** Comparison of ET length and tympanograms in the DS group ET: eustachian tube, DS: Down syndrome, ANOVA: analysis of variance

Tympanogram	N	ET length					One-way ANOVA
		Min	Median	Max	Mean	SD	
A	37	31	40	51	39.65	4.608	P = 0.0426, significant difference
B	8	31	35.5	40	35.63	2.504	
C	5	30	36	44	36.6	5.079	

**Table 3:** Comparison of PUFH length and tympanograms in the DS group PUFH: posterior upper facial height, DS: Down syndrome, ANOVA: analysis of variance

Tympanogram	N	PUFH					One-way ANOVA
		Min	Median	Max	Mean	SD	
A	37	23	44	58	43.84	6.296	P = 0.0332, Significant Difference
B	8	35	37	45	38.25	3.576	
C	5	38	39	45	40	2.915	

**Table 4:** Comparison of sba-PL angle and tympanograms in the DS group s: sella, ba: basion, PL: palatal line, ANOVA: analysis of variance, DS: Down syndrome

Tympanogram	N	sba-PL					One-way ANOVA
		Min	Median	Max	Mean	SD	
A	37	46	60	70	58.19	6.603	P = 0.0007
B	8	42	51.5	56	50.13	4.941	
C	5	45	50	53	49.8	2.95	

**Table 5:** Comparison of sba-ET angle and tympanograms in the DS group s: sella, ba: basion, ET: eustachian tube, ANOVA: analysis of variance, DS; Down syndrome

Tympanogram	N	sba-ET Length					One-way ANOVA
		Min	Median	Max	Mean	SD	
A	37	55	70	85	70.73	5.858	P = 0.0006
B	8	51	62	70	62.13	6.312	
C	5	57	65	70	64.4	4.879	

## Discussion

DS has a relatively high rate of occurrence among genetic disorders and has some definite risk factors like increased maternal age [10]. Karyotyping is the most reliable tool to diagnose DS; an extra chromosome or a genetic material from chromosome 21 can be identified definitively. Although the genetic causes of DS may vary, i.e., mosaicism, translocation, or trisomy 21, DS shows a multitude of classical craniofacial characteristics, such as mongoloid slant, ear abnormalities, epicanthic folds, flat facies and hypotonia [11]. An in-depth understanding of these craniofacial and audiological abnormalities is extremely important due to their high incidence and severity [12]. Early intervention can help reduce the severity of several craniofacial abnormalities, such as upper airway obstruction, obstructive sleep apnea syndrome, deafness, speech delay, and otitis media, which frequently occur in these children in later life.

Being a standardized technique in radiography, cephalometry offers valuable information about the intracranial parameters of growth and development. A few such important and radiologically measurable linear parameters included in this study are ET length, TCB, PUFH, and MD. In normal, healthy adults, the ET, also known as the pharyngotympanic tube has two components, a lateral bony portion attached to the tympanic cavity of the middle ear and a fibro-cartilaginous part that extends into the nasopharynx. The ET runs downward, forward, and medially from the middle ear to the nasopharynx [13]. This natural direction allows for the ET to function as a dynamic conduit, optimize middle ear sound transmission, and provide protection to the delicate structures of the middle and inner ear by providing drainage while also equalizing pressure with the nasopharynx [13]. In the past, there have been some reservations regarding the anatomic points to measure ET [14]. Previously, the point between the posterior border of the maxilla and the anterior border of the PP was considered and not the medial PP that has been recognized as the true localization of the nasopharyngeal end of the ET [14-17]. In our study, the ET length was calculated as the distance from ‘mep’ to ‘pt.’ The various cranial base parameters like the TCB length have been known to influence a number of variables, including the growth of the middle ear, facial and mandibular skeletal development, etc. [18]. The growth and development of a normal child lead to cranial growth accompanied by anterior displacement of the middle cranial fossa, naso-maxillary complex, and the palatal plane. Hence, PUFH and MD are considered important variables to signify cranial growth and remodeling [18-20]. DS classically shows developmental and mental retardation.

In the current study, we observed that the values of all cephalometric measurements in the DS group showed a clear trend to be lower than the values of the normal counterparts (Figures 2-3). To evaluate the audiological parameters, tympanometry was conducted for the controls and the DS group. Tympanometry has emerged as a superior tool for the same due to its high degree of sensitivity, total objectivity, and minimal need for subject cooperation [21]. As expected, all the controls were found to have a normal, ie., type ‘A’ tympanogram (suggests normal middle ear functioning; peak is between +/- 100 daPa; compliance from 0.3 to 1.5 ml). The DS group showed type ‘A’ and ‘B’ (suggests middle ear involvement from fluid (middle ear effusion); there is no identifiable peak; ear canal volume is normal) and type ‘C' (suggests ET dysfunction; often seen just before or after effusion; peak is below 100 daPa; compliance from 0.3 to 1.5 ml (Figure 4) [22]. Hence, we decided to analyze the data to explore any statistical relation between the cephalometric readings among the subjects who presented with type B or C tympanogram in comparison to those with A. Here, we were able to identify a significant result (*p*-value for ANOVA <0.05) from the measurements of two of the linear parameters, i.e., ET length and PUFH length, and both the angular parameters, i.e., s-ba to PL and s-ba to ET length when comparing A, B, and C (see Tables 2-5). Further, the Tukey’s multiple comparison test also detected a '*p*-value' significant for the comparison of A and B; hence, there exists a significant difference in the sba-PL angle and the sba-ET angle between individuals having type A and B tympanograms.

The reduced values of various cranial base dimensions, i.e., ET length, MD, and PUFH, have been positively linked to increased incidences of ear effusion and otitis media previously [23-24]. Since these dimensions are classically lower in DS, patients with cleft palate and brachycephalic adults, they are much more susceptible to chronic ear infections that severely diminish the quality of life. [25-26]. However, we recognize that the current study has its limitations, and a larger sample size along with age- and sex-matched controls should be considered in further studies to reduce the number of confounding variables and obtain results that can be generalizable and applied to a larger population.

## Conclusions

The linear and angular cephalometric readings of the DS group were detected to be less than the normal controls. Although the tympanometric readings of the DS group showed considerable variation, we could identify a relationship between the tympanometric and cephalometric readings. With the emerging advances in medicine, the lifespan of patients with DS has increased, but the quality of life can be improved further. Early diagnosis and timely interventions can help identify patients at risk and take appropriate measures to minimize the impending handicap. In the future, we can suggest the use of a tympanogram, a non-invasive, early intervention tool, to identify the possible deficiencies in cranial measurements. Patients who are recorded to have abnormal tympanometric readings over a period of six months can be subjected to further investigations like a cephalometric analysis.
